# mRNA and DNA selection via protein multimerization: YB-1 as a case study

**DOI:** 10.1093/nar/gkv822

**Published:** 2015-08-13

**Authors:** Dmitry A. Kretov, Patrick A. Curmi, Loic Hamon, Sanae Abrakhi, Bénédicte Desforges, Lev P. Ovchinnikov, David Pastré

**Affiliations:** 1 Laboratoire Structure-Activité des Biomolécules Normales et Pathologiques, INSERM U1204 and Université Evry-Val d'Essonne, Evry, 91025 France; 2 Institute of Protein Research, Russian Academy of Sciences, Pushchino, Moscow Region 142290, Russia

## Abstract

Translation is tightly regulated in cells for keeping adequate protein levels, this task being notably accomplished by dedicated mRNA-binding proteins recognizing a specific set of mRNAs to repress or facilitate their translation. To select specific mRNAs, mRNA-binding proteins can strongly bind to specific mRNA sequences/structures. However, many mRNA-binding proteins rather display a weak specificity to short and redundant sequences. Here we examined an alternative mechanism by which mRNA-binding proteins could inhibit the translation of specific mRNAs, using YB-1, a major translation regulator, as a case study. Based on a cooperative binding, YB-1 forms stable homo-multimers on some mRNAs while avoiding other mRNAs. Via such inhomogeneous distribution, YB-1 can selectively inhibit translation of mRNAs on which it has formed stable multimers. This novel mechanistic view on mRNA selection may be shared by other proteins considering the elevated occurrence of multimerization among mRNA-binding proteins. Interestingly, we also demonstrate how, by using the same mechanism, YB-1 can form multimers on specific DNA structures, which could provide novel insights into YB-1 nuclear functions in DNA repair and multi-drug resistance.

## INTRODUCTION

YB-1 was first described as an mRNA-binding protein expressed in all mammalian tissues to regulate translation (1–3). The functions of YB-1 in translation are most probably essential for mammalian development as YB-1 deficiency in mouse results in prenatal death (4). In the cytoplasm, YB-1 interacts directly and strongly with mRNA (K_D_∼nM, (5,6)) via its single cold-shock domain which possesses two highly conserved RNA recognition motifs (7), RNP-1 and RNP-2, and its unstructured and positively-charged C-terminal tail (8,9). As shown by atomic force microscopy, YB-1 can form multimers in the presence of mRNA and force mRNPs to adopt a beads-on-a-string structure (10). When YB-1 interacts with mRNA close to saturation, the resulting mRNP particles cannot be translated in cell free systems while, well below saturation, YB-1 favors translation (11,12). In line with its role as translation repressor, sucrose gradient analyses of various cell extracts have shown that YB-1 is mostly present in the non-polysomal fraction containing free mRNPs (13). For these reasons, YB-1 is considered as a major component of free mRNPs in the cytoplasm.

However, how YB-1 can exert its function in translation repression while a large amount of YB-1 (about 30 nucleotides per YB-1) is required to stop translation (9,14) remains an unanswered question. Rather than being homogenously distributed among mRNAs, a strong bias for the YB-1 binding to mRNAs may allow its accumulation on specific mRNA transcripts in order to prevent their translation. In line with such biased binding, it has been found that YB-1 orchestrates a selective translational repression in cells (13,15) like that of its own mRNA (16). Despite such YB-1-consuming mechanism, YB-1 can still target a significant number of transcripts in cells. For example, in mouse fibroblasts, about 2 × 10^6^ copies of YB-1 were detected using SILAC technology (17). Therefore, assuming an average mRNA length of 2 × 10^3^ nucleotides (18) and without taking into account the binding of YB-1 to others RNA than mRNA, the amount of YB-1 could be sufficient to repress the translation of at most 3–4 × 10^4^ mRNA molecules. Even overestimated, the YB-1-repressed mRNAs may thus represent a non-negligible fraction of mRNPs if we consider that about 30% of the 2 × 10^5^ mRNAs present in the cytoplasm are in their non-polysomal state, as measured in yeast (19). The preference of YB-1 for A/C-rich hexa/hepta nucleotides (16,20) or other mRNA sequences/structures (21–23) may provide the basis for the selective binding of YB-1 to mRNA. Upon binding to mRNAs containing specific sites, YB-1 could then accumulate on these mRNAs via a cooperative binding. This model thus provides a rational explanation for both YB-1-mediated repression of a specific set of mRNAs and the large amount of this protein per mRNA required to stop translation. In the present work, we explored whether there is a mechanistic model to support such hypothesis.

Another issue addressed in this study is the interaction of YB-1 with DNA as YB-1 is known as a DNA/RNA binding protein (24). Some reports have indeed revealed the translocation of YB-1 to the nucleus and have proposed putative nuclear functions for this protein (25). The nuclear translocation of YB-1 is observed following treatment with some anti-cancer drugs like cisplatin (26), possibly after the cleavage by the proteasome in its C-terminal domain (27) but other mechanisms have also been proposed (28). In the nucleus, YB-1 is present in pre-splicesomes and could participate in exon skipping (29) and regulate RNA splicing (20). In addition, YB-1 may also interact with the promoter regions of genes involved in DNA repair such as topoisomerase IIα (30) to regulate their transcription (31), a function more and more challenged due to the rather unspecific binding of YB-1 to DNA (32,33). The translocation of YB-1 to the nucleus also correlates with multidrug resistance (MDR) (25), which has attracted much interest (34,35) and revealed YB-1 as an important factor for DNA repair (36,37). However, despite its preferential binding to mismatches or damaged DNA (36), the interaction of YB-1 with dsDNA is weak (K_D_∼μM, (38,39)). We may then wonder whether YB-1 can compete with other DNA-binding proteins in the nucleus (40). As explored here, a possible mean for YB-1 to gain access to DNA is not to act as an isolated protein but rather as a multimer in order to effectively compete in the cellular context with other DNA-binding proteins despite its weak affinity to DNA as an isolated protein.

Using an original approach based on single molecule data extracted from atomic force microscopy images (41), we analyzed the relative role of the cold-shock domain and the unstructured C-terminal tail of YB-1 on its interaction with long mRNA and DNA molecules at a nanometric scale. These data combined with other obtained using classical techniques like gel mobility shift assay provide a novel mechanistic view on how YB-1 interacts with both mRNA and DNA. The interest of this approach lies in considering the role of YB-1 multimerization, which could not have been grasped by using short DNA and RNA nucleotides. We then took advantage of our findings to address the concerns raised regarding the selective translational repression mediated by YB-1 and its significant binding to dsDNA structures. We finally discuss the interests of this model to the more general issue of the translation regulation operated by mRNA-binding proteins. In addition, the results provide further insights into the putative role of YB-1 in DNA repair as we discovered its selective targeting of DNA crosses.

## MATERIALS AND METHODS

### Preparation of recombinant proteins

Recombinant YB-1 constructs were expressed in *E. coli* BL21 strain and proteins were purified as described (42,43). Briefly, YB-1 was synthesized in *E. coli* from the pET-3–1-YB-1 plasmid and purified by Heparin-Sepharose, MonoS and Superose 12 (GE Healthcare) chromatography. Fractions containing pure YB-1 were concentrated and dialyzed against 20 mM Hepes-KOH [pH 7.4], 0.5 M KCl. The pET-3–1-YB-1-tr plasmid was provided by Dr. Alexey Sorokin. YB-1-tr was purified the same way as YB-1 except that it was dialyzed against 20 mM Hepes-KOH [pH 7.4], 0.15 M KCl. Plasmids encoding AP-CSD, CSD (Cold-shock domain) and CTD (C-terminal domain) were provided by Dr. Sergey Guryanov. AP-CSD and CSD were isolated by ammonium sulfate fractionation and chromatography using SP-Sepharose, Phenyl-Sepharose and MonoS columns (GE Healthcare). AP-CSD and CSD were stored in 20 mM Hepes-KOH [pH 7.4], 0.2 M KCl or 50 mM potassium phosphate [pH 7.4], respectively. CTD was purified using TALON Metal Affinity Resin (Clontech) and Heparin-Sepharose chromatography. After, CTD was concentrated and dialyzed against 20 mM Hepes-KOH [pH 7.4], 0.5 M KCl. To construct plasmids for CTD1 and CTD2, the corresponding DNA fragments were PCR-amplified and inserted into the pET-22b plasmid. CTD1 and CTD2 were synthesized in *E.coli* and isolated by ammonium sulfate fractionation and chromatography using SP-Sepharose and Superose 12 columns. CTD1 and CTD2 were stored in 20 mM Hepes-KOH [pH 7.4], 0.5 M and 0.15 M KCl, respectively. The DNA fragment corresponding to the mutated YB-1 with two amino acids substitutions in CSD (Y72A F74A) was cloned into pET22b plasmid and purified by the same way as wild type YB-1. Concentrations were estimated from 280 nm absorbance using calculated extinction coefficients (ProtParam tool, http://expasy.org/tools/protparam.html). The OD_280_/OD_260_ ratios were ∼2, thus showing the absence of nucleic acids from the protein samples. Human recombinant G3BP was purchased from Novus Biological. Human PARP1 was a gift from Dr. Maria Sukhanova. The integrity of the recombinant proteins was tested after migration in a denaturating gel (Supplementary S1).

### *In vitro* transcription and capping

Linearized plasmids, pT3Luc, pSP72–2Luc and pcDNA3-HA-YB-1 (provided by Dr. Dmitry Lyabin) were used as templates for RNA synthesis by T7 polymerase of Luc (∼1500 nt), 2Luc (∼3000 nt) and YB-1 mRNA (∼1500 nt), respectively. *In vitro* Transcription was performed by HiScribe T7 High Yield RNA Synthesis Kit (NEB). Short 91 nt RNA was synthesized by the same way using linearized plasmid pBluesript II SK encoding region (from 1339 nt to 1430 nt) of 3′UTR YB-1 mRNA (provided by Alexander Doronin). In order to obtain fluorescent mRNAs, linearized plasmids pcDNA3-HA-YB-1 and pSP72–2Luc were used as templates for RNA synthesis by T7 polymerase (NEB). The transcription mixture was supplemented with fluorescent nucleotides, Atto680-UTP (for 2Luc and Luc mRNAs) or DY776-UTP (for HA-YB-1 mRNA) (Jena Bioscience). Synthesized RNAs were purified using phenol extraction. mRNAs were capped using Vaccinia Capping System according to the manufacturer's recommendations (NEB).

### Gel mobility shift assays

Indicated amounts of YB-1, its fragments and G3BP were incubated with 0.25 pmol of 2Luc mRNA, 0.5 pmol of Luc and YB-1mRNA, 7.5 pmol of 91 nt RNA or 70 fmol of pBR322 (NEB) in 10 μl of binding buffer (20 mM Hepes [pH 7.6], 50 mM KCl, 4 mM MgCl_2_) at 37°C for 10 min. Complexes were separated in 0.75% (2Luc, Luc, YB-1 mRNAs) or in 1.5% agarose gel (91 nt RNA) in 1X TBE buffer for gel shift with RNA and in 1% agarose gel in 1X TAE buffer for gel shift with DNA at room temperature at 25 V for 1 h and were stained with 0.5 μg/mLl EtBr or alternatively using Odyssey Visualizing System (Li-COR) when fluorescent RNAs were used.

### Cross-linking assays

5 μg of YB-1 or its fragments were incubated in the 10 μl of buffer (20 mM Hepes-NaOH [pH 7.6], 50 mM NaCl, 0.5 mM PMSF) containing 0.1% glutaraldehyde for 10 min at room temperature. The reaction was stopped by the addition of 1 μl of 1M Tris-HCl [pH 7.9], and 4 μl of 4X SDS-PAGE sample buffer, and incubation at 95°C for 5 min. Cross-linked proteins were separated by 12% SDS-PAGE and visualized by Coomassie-blue staining.

### Cell culture and transfection

NRK-52E cells (ATCC, Manassas, VA, USA) are rat kidney epithelial cells originating from proximal tubules. They were cultured in Dulbecco's modified Eagle's medium (DMEM) supplemented with 5% (v/v) fetal bovine serum (FBS), 2 mM L-glutamine and 1% antibiotics (penicillin and streptomycin) in a humidified 5% CO_2_ atmosphere at 37°C. Transient transfection of plasmid DNA was performed using Lipofectamine™ 2000 (Invitrogen). The cDNAs encoding the full-length YB-1, YB-1-tr, CSD, CTD, CTD1 and CTD2 were cloned into the XhoI and BamHI sites of the pEGFP-C3 vector (Clontech). The cDNA encoding G3BP was cloned into HindIII and BamHI of the pMRFP-C1 vector (gift from Cecile Gauthier). After transfection, cells were fixed with 4% PFA, 150 mM sucrose in PBS for 20 min at 37°C. For *in situ* hybridization, cell were fixed with 4% PFA (in PBS-sucrose) for 30 min at 37°C, incubated with 100% ice-cold methanol for 10 min at -20°C, in ice-cold 70% ethanol for 10 min at -20°C and then in 1 M Tris (pH = 8) for 5 min. We then added the Cy3-conjugated oligonucleotides (Ploy (T) of 40 nucleotides, Sigma) in the hybridization buffer (0.005% BSA, 1 mg/ml yeast RNA, 10% dextran sulphate, 25% formamide in 2X SSC) at 1 μg/μl. Slides were then placed in a humidity chamber for 1 h at 37°C and shacked. Following hybridization, cells were washed once with 4X SSC and once with 2X SSC. NRK cells were also labeled with anti-YB-1 antibody to detect stress granules.

### Atomic Force microscopy

Ten microliters of each sample were deposited on freshly cleaved mica and dried with a filter paper for AFM imaging. The electrostatic adsorption of both protein:mRNA or protein:DNA complexes on mica was mediated by divalent magnesium cations which are present in the binding buffer (20 mM Hepes [pH 7.6], 50 mM KCl, 4 mM MgCl_2_). All AFM experiments were performed in intermittent contact mode with a multimode AFM instrument (Digital Instruments, Veeco, Santa Barbara, CA, USA) operating with a Nanoscope IIIa controller. We used AC160TS silicon cantilevers (Olympus, Hamburg, Germany) with resonance frequencies of around 300 kHz. The applied force was minimized as much as possible. Images were collected at a scan frequency of 1.5 Hz and a resolution of 512 × 512 pixels. The scan frequency was typically 1 Hz per line, and the modulation amplitude was of about a few nanometers. Statistical analyses were performed using the nanoScope analysis software.

### Topoisomerase assay

Cell extracts with topoisomerase activity were purified as described before with minor modifications (44). Briefly, NRK cells were harvested with PBS-EDTA, washed twice with PBS and resuspended in low-salt buffer (20 mM Tris-HCl [pH 7.6], 5 mM KCl, 1 mM MgCl_2_, 10% Glycerol, 1 mM DTT, 1 mM PMSF). After 10 min incubation on ice, the plasma membrane was disrupted by Dounce homogenizer (narrow pestle) and intact nuclei were collected by centrifugation for 3 min at 15 000 × g. After nuclei were resuspended in high-salt buffer (law-salt buffer containing 0.5 M KCl), incubated for 80 min on ice and centrifuged for 10 min at 25 000 × g. Supernatant was tested for topoisomerase activity and was used in further experiments. Kinetoplast DNA was purchased from Topogen. Standart decatenation reactions (20 μl) contained 300 ng kDNA and in freshly prepared reaction buffer (20 mM Tris-HCl [pH 7.6], 50 mM KCl, 10 mM MgCl_2_, 1 mM ATP, 1 mM EDTA, 1 mM DTT and 30 μg/ml BSA). Reactions were stopped after 30 min by the addition of 5 μl of stop buffer (30% glycerol, 0.5% bromophenol blue and 1% SDS) and resolved on 0.8% agarose gel with 0.5 μg/ml EtBr.

### Purification of proteins bound to poly (A) mRNAs

NRK cells were transfected with the pEGFP-C3 plasmid either empty or encoding different fragments of YB-1 (full-length YB-1, YB-1-tr, AP-CSD and CTD) using Lipofectamine^TM^ 2000. Twenty four hours after transfection, cells were washed twice with 1X PBS and scraped in the lysis buffer (Tris-HCL 20 mM, pH = 8.0, NaCl 150 mM, Triton X100 1% and complete protease inhibitor (Roche). Then, cells were centrifuged for 20 min 16 000 × g at 4°C and pellets were discarded. Supernatants were mixed with Oligo d (T)_25_ Magnetic Beads (NEB) in the lysis buffer and incubated overnight at 4°C. Finally, beads were three times washed with lysis buffer and mRNAs and proteins bound to them were eluted using 1.5X Laemli buffer. Fragments of YB-1 bound to poly (A) mRNAs were detected by western blotting using specific antibodies anti-GFP (Cell Signaling).

### *In vitro* translation assays

Translation occurs in an incubation mixture (30 μl) containing 20 μl of nuclease-treated rabbit reticulocyte lysate (Promega), amino acids (20 μM) and mRNA as indicated in figure legends. Translation was performed at 30°C for the indicated amount of time. New synthetized proteins were visualized by western-blotting using specific antibodies (anti-luciferase, anti-HA).

## RESULTS

### The C-terminal tail of YB-1 is mostly responsible for the binding of YB-1 to mRNA *in vitro* and in cells while the cold-shock domain, by itself, does not significantly bind to mRNA

We first examined at the single molecule level by atomic force microscopy (AFM) the roles of two YB-1 domains on its binding to mRNA: (i) the cold-shock domain of YB-1 which has a β-barrel structure and contains two RNA-binding motifs (7); (ii) the unstructured C-terminal domain of YB-1 which is highly positively charged and thus binds to mRNA. Besides, it may also participate to YB-1 multimerization via the presence of alternate clusters of positive/negative residues (2,45). The alanine/proline rich domain (AP) located at the N-terminus of YB-1, which is not involved in the interaction of YB-1 with RNA (2) but rather with protein partners, is not further considered in this study. Surprisingly, albeit being the most studied, the role of the cold-shock domain in the binding of YB-1 to RNA remains elusive. Indeed, the cold-shock domain alone has a low affinity to mRNA (46) *in vitro* and, in contrast with the Unr protein which displays five cold-shock domains (47), a single cold-shock domain may hardly compete with other mRNA-binding proteins to gain access to mRNA. As previously reported (9), this is rather the highly positively charged C-terminal domain which binds strongly to mRNA via electrostatic interactions.

**Figure 1.**
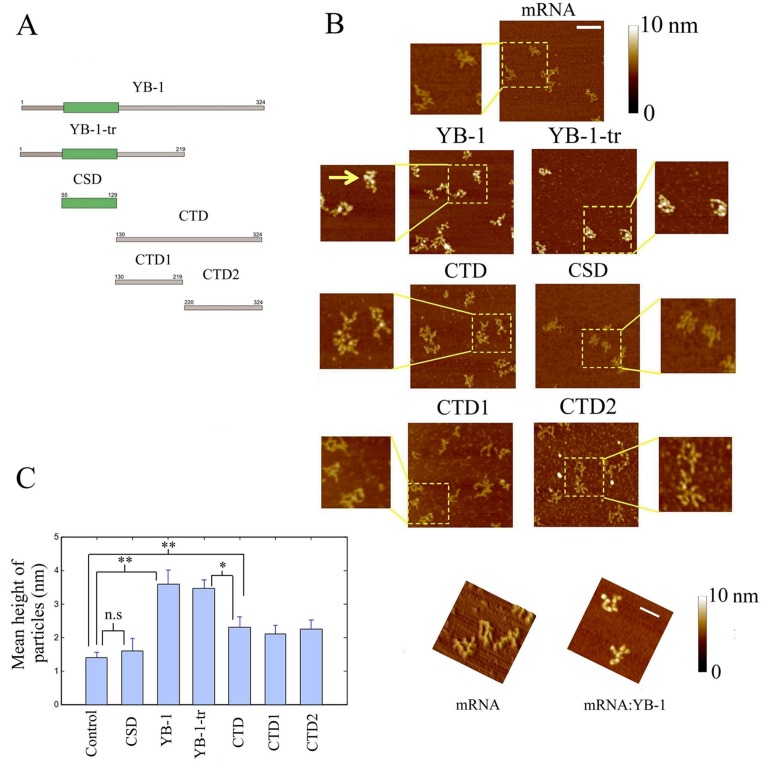
The CTD of YB-1 is required for its binding to mRNA while its cold-shock domain is rather important for the formation of beads-on-a-string structure. (**A**) Schematic view of the YB-1 constructs used in this study. (**B**) The formation of nucleoprotein particles after the interaction of 2Luc mRNA (2 nM) with the YB-1 constructs (300 nM) was detected on a mica surface by AFM, as described in Materials and Methods. We noticed the presence of the typical beads-on-string structures in the presence of both YB-1 (see arrow) and YB-1-tr compared to free mRNA. Scale bar: 200 nm. (**C**) Left panel: Measurements of the particle heights on the mica surface. In the presence of all YB-1 constructs, except the CSD, a significant increase in the particle height compared to free mRNA was measured, which reflects their binding to mRNA. Due to the formation of beads-on-a string structures, the mean height of the particles was however larger in the presence of YB-1 and YB-1-tr than in the presence of the CTD, CTD1 and CTD2. The CSD may therefore be necessary for the formation of the beads-on-a-string structures but not for the binding to mRNA. The ‘particle analysis’ application of the Nanoscope IIIa software (version 5) over at least 50 particles was used to measure the heights on three different samples. Results are mean ± SD. *P < 0.05; **P < 0.01; by *t*-test. Right panel: typical AFM images in 3D showing the marked difference between the branched structure of free mRNA and the beads-on-a-string-structures of saturated YB-1:mRNA complexes. Scale bar: 200 nm.

In order to reveal the respective roles of the CSDs and CTDs, we analyzed the binding to mRNA of several YB-1-derived constructs (Figure 1A): YB-1, full length; YB-1-tr, a truncated form comprising the first 219 amino acid residues which mimics the cleaved form of YB-1 by the 20S proteasome after stress (27); CSD, the cold-shock domain, or AP-CSD (AP+CSD domains), both giving the same results regarding their interaction with mRNA; CTD, the entire C-terminal domain; CTD1 and CTD2, two halves of the C-terminal domain which are separated following the 20S proteasome cleavage. So far, only the structure of full length YB-1:mRNA complexes was analyzed by AFM (10). For this analysis, we used a synthetic 2Luc mRNA of about 3000 nucleotides, which allows a better visualization of the YB-1:mRNA structure than shorter mRNA. A single YB-1 binds to about 30 ribonucleotides. At saturating YB-1 concentration (YB-1/2Luc mRNA molar ratio of 150), isolated mRNPs with typical beads-on-a-string structure were clearly detected by AFM (Figure 1B and C), in line with a previous report (10). A similar pattern was also observed for YB-1-tr after its interaction with mRNA. At the same molar ratio, the binding of CTD, CTD1 and CTD2 with mRNA could be also detected but, interestingly, CTD, CTD1 and CTD2 alone cannot induce the formation of beads-on-a-string structures. In contrast with the other constructs, the binding of CSD to mRNA was not detected by AFM under such conditions, which should be due to its weak affinity to mRNA.

**Figure 2.**
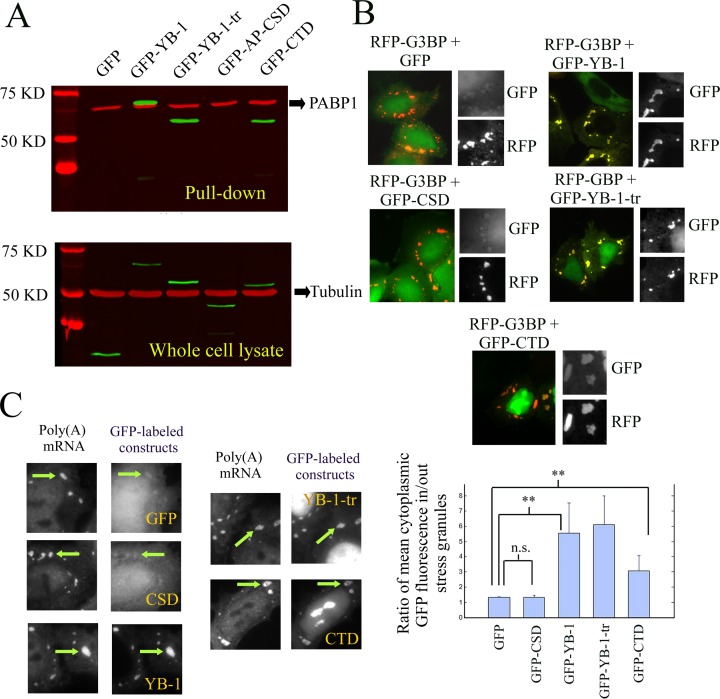
Putative binding of YB-1, YB-1-tr, CTD and CSD to mRNA as tested by pull down assays or by detecting their presence in stress granules. (**A**) Western blot analysis of pull down assays from whole cell lysate of NRK cells expressing GFP-labeled YB-1 constructs. Oligo (T)_25_ magnetic beads were used to isolate Poly (A) mRNA and its protein partners. The GFP-labeled constructs were then detected using anti-GFP antibody (in green). Tubulin was used as a loading control for the whole cell lysates (anti-tubulin antibody in red). As revealed with anti-PABP1 antibody, the presence of PABP1 in the pull down fraction confirmed the effectiveness of the pull down assay to isolate Poly (A) mRNA. AP-CSD was the only YB-1 construct undetected in the pull-down fraction. (**B**) NRK cells were co-transfected with plasmids coding for one of the GFP-labeled YB-1 constructs and RFP-G3BP. The presence of the YB-1 constructs in G3BP-induced stress granules reveals their cellular affinity for mRNA. The presence of YB-1, YB-1-tr and CTD was clearly detected in stress granules (see arrows) in contrast with the CSD, which displays a similar distribution that GFP, used as control. Scale bar: 30 μm. Lower panel: Analysis of the relative enrichment of the various YB-1 constructs in stress granules. We measured the ratio of the relative increase of the mean intensity of GFP-labeled YB-1 constructs in stress granules compared to the surrounding cytoplasm. Such increase was significantly different compared to GFP for YB-1, YB-1-tr and CTD but not for CSD. Results are mean ± SD over 3 different samples, 10 cells per samples and 3 stress granules per cell. *P < 0.05; **P < 0.01; by *t*-test. (**C**) NRK cells were transfected with plasmids encoding for the indicated GFP-labeled YB-1 constructs and exposed to 0.3 mM arsenite for 45 min. *In situ* hybridization was then performed to reveal Poly (A) mRNA with Cy3-conjugated oligo(dT)_40_. Scale bar: 10 μm.

We then explored whether the interactions of the various YB-1 constructs with mRNA detected *in vitro* by AFM are relevant in a cellular context using NRK cells transfected with plasmids encoding for the YB-1 constructs fused to a N-terminal GFP tag. To that end, we used oligo(dT)_25_ magnetic beads to isolate, from NRK cell extracts, poly (A) mRNA along with mRNA-binding proteins and other proteins indirectly interacting with mRNA (Figure 2A (48)). The results clearly indicate that GFP-YB-1, GFP-YB-1-tr and GFP-CTD probably interact with mRNA. In contrast, GFP-AP-CSD was not detected in the pull down fraction.

To further probe the affinity of YB-1 for mRNA but without disrupting the cell membrane, we took advantage of the spatial enrichment of mRNA in stress granules (49,50). Stress granules are non-polysomal mRNA-containing aggregates of micrometric size that appear in the cytoplasm of mammalian cells, either upon acute stress conditions (51) or after the expression of pro-aggregating RNA-binding proteins like TIA-1 or G3BP (52). The presence of YB-1 in stress granules can either be due to its binding to mRNA, owing to its high affinity for mRNA, or to its binding to other proteins found in stress granules, which cannot be excluded. In NRK cells expressing RFP-G3BP, we found that GFP-YB-1, GFP-YB-1-tr and GFP-CTD significantly accumulate in stress granules compared to GFP alone (Figure 2B and C). On the other hand, GFP-CSD was not found enriched in stress granules, its spatial distribution being similar to that of GFP alone. The affinity of CSD for mRNA is probably not sufficient to allow its accumulation in stress granules.

Due to the low affinity of CSD for mRNA, we then considered an alternative role for CSD and our attention turned to a putative role in a cooperative binding to mRNA since, in bacterial cold-shock proteins, the cooperative binding to RNA has been reported (53,54).

### The cold-shock domain of YB-1 is critical for the cooperative binding of YB-1 to mRNA

**Figure 3.**
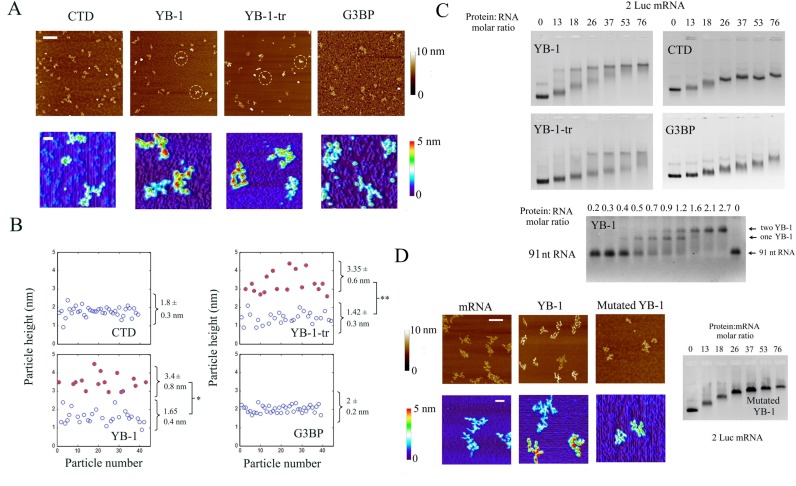
YB-1 binds to mRNA in a cooperative manner via its cold-shock domain. (**A**) The formation of nucleoprotrein complexes in the presence of 2 Luc mRNA (2 nM) and the YB-1 constructs at concentration below saturation (50 nM) was analyzed by AFM on mica surface. Upper image: Large scale AFM images reveal an inhomogeneous binding of YB-1 and YB-1-tr to mRNA in contrast with CTD and G3BP used as control mRNA binding protein. Dashed circles indicate the presence of putative saturated mRNA:YB-1 complexes, which appear brighter than the other particles due to their height. Scale bar: 300 nm. Lower panel: The non-homogenous binding of YB-1 and YB-tr, which indicates a cooperative behavior, can be easily observed on higher magnification images. We also noticed the presence of the typical beads-on-a-string for the saturated complexes. Scale bar: 50 nm. (**B**) Analysis of the height distribution of the particles on mica. In contrast with CTD and G3BP, two significantly different populations can be extracted from the data obtained after the interaction of mRNA with YB-1 and YB-1-tr (see Figure S5). The population with larger (filled circles) and lower (empty circles) heights correspond to saturated YB-1:mRNA complexes and free or unsaturated mRNA, respectively (see Figure 1). Results are mean ± SD. *P < 0.05; **P < 0.01; by *t*-test. (**C**) Gel mobility shift assay reflecting the binding of CTD, YB-1, YB-1-tr and G3BP to 2 Luc mRNA and the binding of YB-1 to a short 91-bp RNA. At intermediary YB-1and YB-1-tr:2Luc mRNA molar ratios (from 13 to 37), the formed complexes split in two distinct bands in the gel corresponding to saturated YB-1:mRNA complexes and free or unsaturated mRNA, which indicates a cooperative binding of YB-1 to mRNA. Such cooperative binding is not apparent for CTD and G3BP as a progressive reduction of the nucleoprotein complex mobility is rather observed. For a short RNA (91 nt), we observe a step-by-step binding of YB-1, as shown by arrows. (See material and methods for experimental conditions of gel mobility shift assay). The binding of AP-CSD was not detected after gel mobility shift assays, which again indicates the low affinity of CSD for mRNA (Figure S8B). (**D**) Right panel: A gel mobility shift assay was performed to probe the binding of YB-1 to 2Luc mRNA after mutations of its cold-shock domain (Y72A and F74A). The binding of the YB-1 mutant appears to be non-cooperative. Left panel: The structure of 2 Luc mRNA (2 nM) in the presence or absence of wild-type or mutant YB-1 (50 nM) was analyzed by AFM on mica surface. In contrast with wild-type YB-1, we failed to observe the typical beads-on-a-string structure with the mutated YB-1. Scale bars: 150 and 50 nm for the highest and lowest magnifications, respectively.

To capture a putative cooperative binding of YB-1 to mRNA, we analyzed its interaction with 2Luc mRNA at the single molecule level by AFM and at a YB-1 concentration well below the saturation level (YB-1:2Luc mRNA molar ratio equals to 25). This method was previously developed to detect the cooperative binding of ssDNA-binding proteins to ssDNA (41). We rapidly noticed a striking inhomogeneous repartition of YB-1 among mRNA molecules (Figure 3A). Some mRNA formed typical beads-on-a-string structure as observed at saturating YB-1 concentration, while others mRNA appeared as free or in complex with few YB-1 molecules (Figure 3B). A cooperative binding of YB-1 to mRNA was also detected by AFM for YB-1-tr but not for CTD. Interestingly, the cooperative behavior observed for YB-1 is not shared by another mRNA-binding protein, G3BP, used as control (Figure 3A and B).

To ensure that the cooperative binding of YB-1 really takes place in the bulk solution and not only on the mica surface used for AFM, we analyzed its binding to mRNA by gel mobility shift assay (Figure 3C). At YB-1 concentrations below saturation, we noticed the appearance of two distinct bands in the gel which should result from the coexistence of saturated YB-1:mRNA complexes and of free or unsaturated mRNA, a typical feature of cooperative behavior. As known for proteins that bind to single-stranded DNA cooperatively (41), increasing the ionic strength reduces the cooperative binding of YB-1 to mRNA (Figure S2A). In contrast with YB-1, even at moderate ionic strength, G3BP binds to mRNA in a non-cooperative manner as indicated by the single migration band of the G3BP:mRNA complex and the progressive reduction of its electrophoretic mobility with increasing concentration of G3BP. Crosslinking experiments further indicate that mRNA serves as a scaffold for the multimerization of YB-1 and demonstrate YB-1 multimerization on mRNA via direct YB-1:YB-1 interactions (Figure S2B).

As both YB-1 and YB-1-tr bind to mRNA cooperatively, two domains, CSD and CTD1, may be involved in the molecular mechanism leading to cooperativity. CTD1 contains two clusters of positive charges (+ 6 e^−^ for 11 residues and +10 e^−^ for 20 residues) and one cluster of negative charges of low charge density (- 4 e^−^ for 14 residues, see Figure S3). The many positive charges of CTD1 can hardly lead to an electrostatic self-attraction of CTD1 but rather to a strong self-repulsion. In line with this, CTD alone fails to display a cooperative binding to mRNA (Figure 3C). Albeit we cannot exclude the participation of CTD1, CSD probably play a central role in this cooperative binding process because both bacterial and mammalian CSD domains bind to RNA cooperatively (53–55). To explore this hypothesis, we analyzed protein multimerization in the absence of RNA after glutaraldehyde exposure and found a significant crosslinking between CSD but not between CTD molecules (Figure S4A) and obtained similar results by AFM (Figure S4B). The positive charges of YB-1 and YB-1-tr located in CTD and CTD1, respectively, most probably hinder multimerization owing to an electrostatic repulsion. Along with this, in 2M LiCl, YB-1 can form long fibrils and it was proposed that CSD plays a key role in the genesis of such fibrils (42).

To further examine the role of CSD in the cooperative binding of YB-1 to mRNA, we generated two point mutations on residues implicated in the binding of CSD to RNA (Y72A/F74A, (56)) and analyzed the binding of the double YB-1 mutant to mRNA by using gel mobility shift assays (Figure 3D). In contrast to YB-1, we observed a single migration band which indicates that the binding of the mutant protein to mRNA is still effective but no longer cooperative. In addition, high resolution imaging by AFM reveals the disappearance of the beads-on-a-string structures typical of saturated YB-1:mRNA complexes, which adds credits to the role of CSD in the cooperative binding of YB-1.

Interestingly, the cooperative binding of YB-1 evidenced for long mRNA was not observed for short 91 nt RNA (Figure 3C). YB-1 protein reduces the mobility of the short RNA via discrete steps typical of a non-cooperative behavior. As for many polymerization processes, a nucleus of critical size needs to be form prior to elongation. The two or three YB-1 which can bind to 91 nt RNA may not be sufficient to form a stable nucleus. This result demonstrates that long RNA should be used to probe the cooperative binding of YB-1 to mRNA.

In summary, we propose that CTD mostly participates in the binding of YB-1 to mRNA through its clusters of positive charges. When YB-1 is bound to mRNA, most of the CTD positive charges are neutralized by the negative charges of mRNA, which prevents the electrostatic self-repulsion of YB-1. The YB-1 self-attraction can then trigger the cooperative binding of YB-1 on mRNA via the CSD.

### The cooperative binding of YB-1 to mRNA can take place in the presence of protein competitors

**Figure 4.**
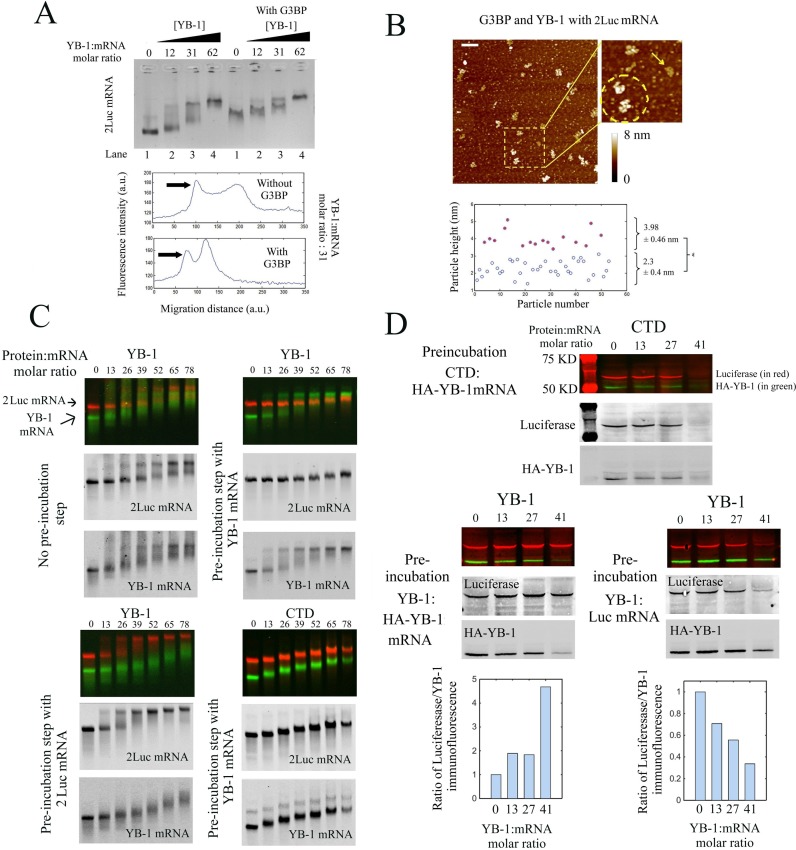
The cooperative binding of YB-1 to mRNA allows a selective translational repression and takes place even in the presence of another mRNA-binding protein, G3BP. (**A**) Upper panel: Gel mobility shift assay probing the binding of YB-1 to 2Luc mRNA in the absence or presence of G3BP (G3BP:2 Luc mRNA molar ratio equals to 50). For a YB-1:mRNA molar ratio between 31 and 62, two bands in the gel are detected which reflects the cooperative binding of YB-1 in the presence of G3BP. Lower panel: Integrated fluorescence intensity of ethidium bromide versus the migration distance with or without G3BP extracted from the upper panel. YB-1:2Luc mRNA molar ratio: 31. In the presence of G3BP, two bands were clearly observed. (**B**) Higher panel: The nucleoprotein complexes formed after the interaction between 2Luc mRNA (4 nM) with an equimolar mixture of G3BP and YB-1 (50 nM each) were analyzed on mica surface by AFM. A non-homogenous binding of YB-1 to mRNA is detected on the mica surface, as shown on the higher magnification image (dashed circle: two saturated YB-1:mRNA complexes, arrow: unsaturated complex). Scale bar: 200 nm. Lower panel: Analysis of the particle heights reveals two distinct populations, which could indicated the coexistence of saturated YB-1:mRNA complexes (3.98 ± 0.46 nm) and mRNA:G3BP complexes (2.3 ± 0.4 nm). (**C**) Gel mobility shift assays in the presence of a mixture of fluorescently labeled 2Luc mRNA (∼3000 nt, 0.25 pmoles, red label) and YB-1 mRNA (∼1500 nt, 0.5 pmoles, green label) at varying concentrations of YB-1 or CTD. These assays were performed after mixing the two mRNAs for 15 min with or without a pre-incubation step. During the pre-incubation step, YB-1 or CTD was allowed to interact with 2Luc or YB-1 mRNA with for 15 min, as indicated in the figure panel. Both YB-1 and 2Luc mRNAs split in two distinct bands in the gel in the presence of YB-1 without the pre-incubation step, which indicates a cooperative binding of YB-1 to both mRNAs. On the other hand, when a pre-incubation step is performed, saturated YB:1mRNA complexes are solely detected for the mRNA that has been pre-incubated with YB-1, whatever it is YB-1 or 2Luc mRNA. The pre-incubation step of CTD with YB-1 mRNA failed to induce a preferential binding of YB-1 to its own mRNA. A homogenous reduction of the electrophoretic mobility for the two mRNAs is rather observed as CTD concentration increases. (**D**) *In vitro* translation assays in rabbit reticulocyte system of an equimolar mixture of Luc mRNA (∼1500 nt, 0.25 pmoles) and HA-YB-1 mRNA (∼1500 nt, 0.25 pmoles) at varying concentrations of YB-1 or CTD. HA-YB-1 or Luc mRNAs were pre-incubated for 15 min with either YB-1 or CTD, as indicated in the figure panel. The two mRNAs were then mixed and added to the reticulocyte extracts for 15 min for *in vitro* translation. The pre-incubation step with YB-1 leads to the preferential translation repression of the pre-incubated mRNA compared to the other mRNA. In contrast, CTD leads to an equal translation repression for both mRNAs despite the pre-incubation step. Each experiment was performed in duplicate. We also tested that the amount of protein synthesized depends on the amount of added mRNA under conditions tested. Lower panel: Plot of integrated immuno-fluorescence intensity of the bands related to luciferase and HA-YB-1 for lanes 1–6. Anti-HA and anti-luciferase antibodies were used to detect newly synthetized HA-YB-1 and luciferase, in green and red, respectively. In supplementary Figure S6B, anti-YB-1 antibody was used to control the total YB-1 concentration for the conditions tested (endogenous, exogenous and HA-YB-1).

At the light of these results, we asked the question as to whether the cooperative binding of YB-1 to mRNA occurs in the presence of competitors present in the cytoplasm. Indeed many other mRNA-binding proteins may compete with YB-1 for the binding to mRNA and may thus hinder the proposed mechanism of cooperative binding. To examine this point, we chose G3BP for the following reasons: (i) G3BP is a cytoplasmic mRNA-binding protein like YB-1 (52); (ii) Our data show that G3BP binds to mRNA in a non-cooperative manner, in contrast with YB-1 (Figure 3C). We therefore analyzed the binding of YB-1 to preformed G3BP:mRNA complexes by gel mobility shift assay and observed the appearance of two bands in the presence of YB-1 below saturating concentration (Figure 4A). The presence of two bands indicates that YB-1 may bind to mRNA cooperatively even in the presence of G3BP. To ascertain this result, we performed a statistical analysis of the AFM data at the single molecule level under similar conditions. The saturated YB-1:mRNA complexes can be easily distinguished owing to their height larger than that of G3BP:mRNA complexes. Our measurements then reveal the presence of two different populations in the presence of YB-1 below saturating concentration which is typical of the cooperative binding of YB-1 to mRNA (Figure 4B and S5). The presence of G3BP is therefore not an obstacle for the cooperative binding of YB-1 to mRNA. A mechanism to account for this result is advanced in the discussion.

### Saturated YB-1:mRNA complexes are stable when mixed with free mRNA which provides a basis for a selective translational repression

To decipher whether YB-1 can target specific mRNAs and thus selectively repress their translation, YB-1 was added to an equimolar mixture of two fluorescent mRNAs, YB-1 and 2Luc mRNAs, which are considered as specific and non-specific YB-1 targets, respectively (16). However, by gel mobility shift assays, we failed to evidence a preferential binding of YB-1 to its own mRNA (Figure 4C). *In vitro*, the use of long and naked mRNA with many non-specific nucleotide sequences may hinder the selective binding of YB-1 to specific RNA sequences. Other protein factors may also come into play to orient YB-1 multimerization on specific mRNAs.

To document whether YB-1 multimerization on specific mRNAs can provide a basis for mRNA selection, we designed a dedicated protocol. One of the two mRNAs, either 2Luc or YB-1 mRNA, was allowed to interact with YB-1 in a pre-incubation step before mixing the two mRNAs together for 15 min. As observed by gel mobility shift assays (Figure 4C), YB-1 forms saturated complexes exclusively with the mRNA that has been pre-incubated with it whatever it is YB-1 or 2Luc mRNA. On the other hand, when YB-1 was replaced by CTD, which does not bind to RNA cooperatively, we rather observed a homogenous and gradual reduction of the electrophoretic mobility for the two mRNAs despite the pre-incubation step. These results indicate that the YB-1 molecules are hardly released from mRNA once saturated YB-1:mRNA complexes have been formed. The energy benefit due to the cooperative binding of YB-1 to mRNA is probably an obstacle to the release of YB-1 from mRNA.

These results prompted us to further explore whether a selective multimerization of YB-1 on mRNAs can indeed lead to a preferential translation repression *in vitro* in rabbit reticulocyte system. As previously described, YB-1 inhibits translation *in vitro* at the initiation step and the CTD alone is sufficient to inhibit translation (9). After pre-incubating YB-1 with Luc or YB-1 mRNA, the two mRNAs were mixed together before being added to rabbit reticulocyte lysate for translation (Figure 4D). We clearly noticed a selective translational repression of the mRNA that has been pre-incubated with YB-1, whatever it is Luc or YB-1 mRNA. As a control, a selective translational repression was not observed in the absence of the pre-incubation step (Figure S6A). Furthermore, even with a similar pre-incubation step, the translation inhibition mediated by CTD is non-selective (Figure 4D). Altogether, these results indicate that the cooperative binding of YB-1 to mRNAs leads to the coexistence of untranslatable mRNAs, which have formed saturated and stable mRNA-YB-1 complexes, and translatable mRNAs, which are free or in complex with few YB-1. The cooperative binding of YB-1 to mRNA could thus provide a basis for a selective translational repression.

### YB-1 binds preferentially to supercoiled DNA at DNA crosses

**Figure 5.**
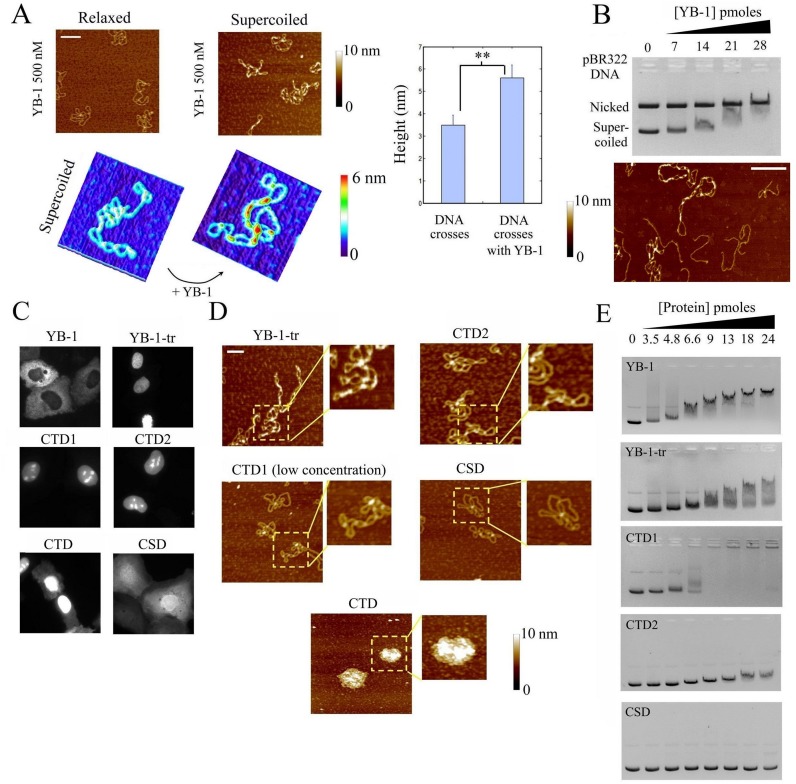
YB-1 binds preferentially supercoiled DNA at crosses (**A**) Left panel: The interaction of YB-1 (500 nM) with nicked or supercoiled pBR322 DNA (2 nM) was probed by AFM on mica. YB-1 forms short multimers on supercoiled pBR322 DNA, especially at DNA crosses, while the binding of YB-1 to relaxed plasmids was not detected. Scale bar: 200 nm. Right panel: Analysis of the heights at DNA crosses of supercoiled DNA in the presence or absence of YB-1. The significant increase in height of DNA crosses in the presence of YB-1 reveals its multimerization at crosses. (**B**) Upper panel: Gel mobility shift assay probing the binding of YB-1 to an equimolar mixture of supercoiled and nicked pBR322 DNA (70 fmoles each). A significant decrease in the mobility of supercoiled DNA occurs at lower concentration than for relaxed plasmid. Two non-exclusive explanations can be advanced for this: (i) YB-1 binds to both supercoiled and relaxed DNA but only reduces the mobility of supercoiled DNA by modifying its conformation, (ii) YB-1 preferentially binds to supercoiled DNA. AFM data (see lower panel) indicate that YB-1 indeed preferentially binds to DNA crosses. Lower panel: AFM image of linearized and supercoiled pBR322 DNA (1 nM each) after their incubation in the presence of 500 nM YB-1. We observed that YB-1 preferentially binds to supercoiled DNA at DNA crosses in the presence of linear DNA. Scale bar: 150 nm. (**C**) Cellular location of the indicated GFP-labeled YB-1 constructs after their expression in normal rat kidney cells. YB-1-tr, CTD, CTD1, CTD2 were rather located in the nucleus in contrast with the cytoplasmic location of YB-1 and the homogenous distribution of CSD. (**D**) The presence of the indicated YB-1 constructs on supercoiled pBR322 DNA (2 nM) was probed by AFM. YB-1-tr (500 nM) form short multimers at DNA crosses in contrast with CTD1 (100 nM) and CTD2 (500 nM). CTD (500 nM) and CTD1 (500 nM, not shown) lead to the formation of mRNA-containing granules. In the presence of CSD, no interaction with supercoiled DNA was observed. Scale bar: 100 nm. (**E**) Gel mobility shift assay representing the binding of the indicated YB-1 constructs with supercoiled pBR322 DNA concentration. In agreement with the AFM results (**D**), a significant shift was detected with YB-1-tr. The shift is less marked for CTD2 and for CTD1 than YB-1 and YB-1-tr, before aggregation takes place. No shift was detected with CSD in the conditions explored here.

YB-1 is a dual RNA/DNA-binding protein. However, so far, no information about the structure of DNA:YB-1 complexes at the molecular level has been reported. As expected (38,39), we found that YB-1 binds to DNA with a low affinity compared to mRNA since no interaction with DNA was detected by AFM at YB-1 concentrations previously used to probe mRNA:YB-1 interactions (50 nM). We therefore used a higher YB-1 concentration (500 nM) to examine its interaction with DNA and probed the presence of YB-1 on both relaxed and supercoiled DNA. The point is to observe whether the affinity of YB-1 to DNA was regulated by the DNA topology. DNA topology should be considered while investigating DNA:protein interaction because of its critical impact on DNA recognition and processing in cells. Interestingly, we found that YB-1 binds much more efficiently to supercoiled than to relaxed DNA as shown by AFM (Figure 5A). In addition, we observed that YB-1 forms multimers along supercoiled DNA which are mostly, if not exclusively, located at DNA crosses. We also examined the selective binding of YB-1 and YB-1-tr to DNA using a mixture of linear and supercoiled DNA and compared the results with those obtained with G3BP used as control mRNA-binding protein. The results were striking: YB-1 multimers were barely detected on linear DNA but most often on supercoiled DNA, which confirms a selective YB-1 multimerization based on nucleation at DNA crosses (Figure 5B and S7). YB-1-tr displays the same selectivity toward DNA crosses as YB-1, as expected owing to its preserved cooperative binding. On the other hand, no significant binding or multimerization of G3BP on supercoiled DNA was observed.

The selective binding of YB-1 to supercoiled DNA was further demonstrated in a competitive gel mobility shift assay performed in the simultaneous presence of nicked and supercoiled DNA. Indeed, we clearly observed the disappearance of the band related to free supercoiled DNA while, at the same YB-1 concentrations, the intensity of the band corresponding to free nicked plasmid remained unchanged (Figure 5B). To further address the possible multimerization of YB-1 at DNA crosses, we finally performed crosslinking experiments which show that YB-1 indeed forms multimers on supercoiled DNA via direct YB-1:YB-1 interactions since they are resistant to DNAse once formed (Figure S2B).

To decipher which domains of YB-1 is responsible for its binding to DNA crosses and for the ensuing YB-1 multimerization, we probed the affinity of different YB-1 constructs to supercoiled DNA by AFM and gel mobility shift assay. YB-1-tr is of special interest because it mimics a possible YB-1 cleavage by the 20S proteasome which induces its nuclear translocation, as observed in transfected NRK cells (Figure 5C). CTD, CTD1 and CTD2 were also partially located in the nucleus, which is probably due their positive charges. The AFM results show that YB-1-tr can form multimers on supercoiled DNA, which are especially located at DNA crosses (Figure 5D), as in the case of full length YB-1. In addition, we detected a significant electrophoretic shift of the migration band corresponding to supercoiled DNA in the presence of YB-1-tr (Figure 5E). In contrast, the interaction of CSD with supercoiled DNA was not detected by AFM or gel mobility shift assay, in agreement with its weak affinity for DNA (7). In the presence of CTD, we detected the presence of large DNA aggregates by AFM, which is probably due to an electrostatic mechanism by considering its clusters of positive charges (Figure S3). Most probably for a similar reason, DNA aggregation could also occur in the presence of CTD1, as observed by gel mobility shift assay (Figure 5E). At CTD1 concentration sufficiently low to prevent DNA aggregation, no multimeric structure was detected on supercoiled DNA by AFM. CTD2, which is globally less charged than CTD1, does not form DNA aggregates. We noticed that CTD2 tends to localize at DNA crosses and also prefers to bind to supercoiled rather than relaxed DNA (Figure S8A), which may be attributed to the electrostatic benefit provided by forming salt bridge at the interface between two crossing DNA helices. On the other hand, in the presence of CTD2, we failed to detect long-ranged multimerization starting from DNA crosses by AFM. In line with this, the migration shift of the supercoiled DNA band is weak and displays little smear with CTD2 compared to what is observed for YB-1-tr or YB-1 (Figure 5E).

In summary only YB-1 and YB-1-tr have the capacity to multimerize at DNA crosses most probably because of the CSD self-attraction. The CTD positive charges preferentially localize at the interface between two crossing DNA helices to form a ‘salt bridge’ and this probably participates to the higher affinity of YB-1 for crosses.

### YB-1 multimerization at DNA crosses leads to the self-alignment of the two facing DNA

**Figure 6.**
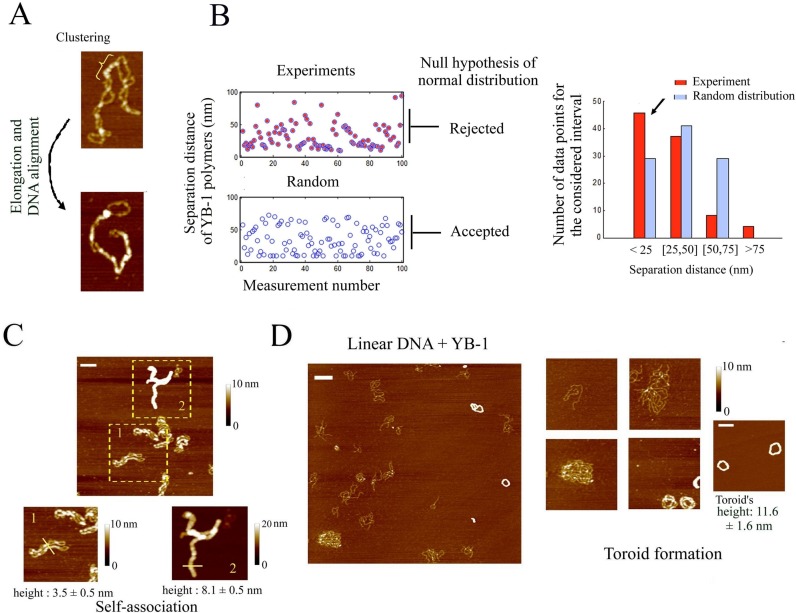
YB-1 multimerization at DNA crosses leads to the alignment of the two interacting DNA helices and higher-order assembly of DNA molecules. (**A**) Clusters of YB-1 (300 nM) multimers are clearly detected on supercoiled DNA (2 nM) and are especially located at DNA crosses (upper image). When many YB-1 multimers are bound to supercoiled DNA, the molecule is forced to adopt a linear shape (lower image). Scale bar: 50 nm. (**B**) When the trajectory adopted by DNA helices is clearly distinguished by AFM all along supercoiled DNA, we measured the separation distance between two consecutive YB-1 multimers along DNA. We then plotted a theoretical curve of a random distribution using the same number of data and the same length of DNA. Separation distances smaller than 10 nm were discarded to mimic the limit imposed by the AFM resolution. We then tested the null hypothesis of a normal distribution for these two cases using the Chi-square goodness-of-fit test at the 5% significance level. In contrast with a random distribution, the null hypothesis was rejected for the experimental results. In agreement with this, the bar plot indicates a higher occurrence of YB-1 which are close from each other (<25 nm) (see arrow). (**C**) Supercoiled DNA (2 nM) were assembled into high order structures on mica in the presence of YB-1 at high concentration (1 μM), as observed by AFM. In the lower right panel, the height of the nucleoprotein structure of cylindrical shape indicates that at least two supercoiled DNA are bundled. The lower left panel shows an intermediary structure of DNA bundles with two supercoiled DNA which are self-aligned. Scale bar: 150 nm. (**D**) YB-1 at high concentration (1 μM) leads to the alignment of 1220 bp linear DNA into large circular structure on mica which, at the end of the process, formed perfect toroids, as observed by AFM. The height of the toroids indicates that many DNA are bundled. Interestingly, free 1220 bp linear DNA coexists with toroids on mica, which suggests a cooperative binding of YB-1 to DNA. Scale bars: 150 nm.

As DNA crosses appear as preferred sites for the binding of YB-1, we explored whether YB-1 may lead to a higher-order assembly of DNA via its multimerization in between two DNA helices. We indeed noticed in AFM images a forced alignment of two interacting DNA helices in the presence of YB-1 multimers (Figure 6A). In addition, we measured by AFM that YB-1 multimers are not homogenously distributed along DNA and tend to be closer to each other than expected from random positioning (Figure 6B). When the YB-1 multimers are close to each other along supercoiled DNA, DNA helices are forced to adopt a parallel trajectory, which increases the occurrence of forming novel YB-1 multimers. In line with this, two parallel supercoiled DNA can be also associated into a single large filament thus indicating the YB-1 multimers can bridge two DNA helices from two different DNA molecules (Figure 6C).

To further ascertain that YB-1 multimerization takes place at the interface between two DNA helices and forces them to adopt a parallel trajectory, we examined the consequences of YB-1 multimerization on the high-order assembly of linear DNA, which has no topological constraint imposed by supercoiling and thus can theoretically be perfectly aligned through YB-1 multimerization. In the presence of YB-1 at high concentration (1 μM) to compensate for the lower affinity of YB-1 to linear than supercoiled DNA, we observed the presence of YB-1 multimers in structures that appear circular and in which many linear DNA tend to adopt a parallel trajectory (Figure 6D). We also noticed the scarce presence of YB-1 multimers on isolated DNA in contrast to its high concentration in multimolecular DNA structures. An inhomogeneous distribution of YB-1 on DNA further evidences the cooperative binding of YB-1 to nucleic acids either RNA or DNA. Interestingly, the circular structure made of many linear DNA molecules can also be compacted into regular toroids via YB-1 multimerization (Figure 6D).

In summary, YB-1 displays a striking affinity at the interface between two DNA crosses which serves as seeds for the cooperative binding and multimerization of YB-1 between two DNA helices.

### YB-1 blocks topoisomerase II activity from NRK cell extracts

**Figure 7.**
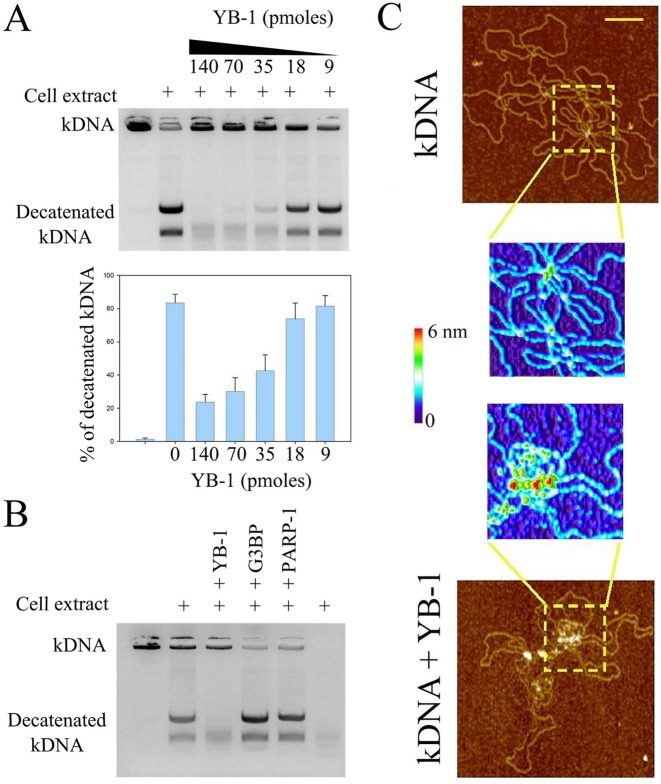
YB-1 inhibits the decatenation of kDNA mediated by topoisomerase II by masking DNA knots (**A**) Topoisomerase II activity from nuclear cell extract was analyzed using kinetoplast DNA (kDNA) decatenation. kDNA which contains catenated DNA circles cannot enter in the gel during electrophoresis. The results of kDNA decatenation by topoisomerase II from the cell extract (NRK cell nuclei) appear as two bands on the agarose gel. YB-1 clearly inhibits the topoisomerase II activity rather abruptly as shown in the histogram which represents the relative integrated intensity of the ethidium bromide fluorescence coming from decatenated kDNA divided by the total ethidium bromide fluorescence of kDNA (catenated plus decatenated kDNA). kDNA: 300 ng per well. Results are mean ± SD over three experiments. (**B**) Topoisomerase II activity from nuclear cell extract in the absence or presence of YB-1, G3BP or PARP-1. Only YB-1 induces a significant inhibition of the topoisomerase II activity. Protein amount: 35 pmoles per well. kDNA: 300 ng per well. (**C**) kDNA was observed on mica by AFM in the absence or presence of YB-1 (1 μM). The higher magnification images show that YB-1 is located at DNA knots. kDNA: 2 μg/ml. Scale bar: 200 nm.

The specific targeting of YB-1 to DNA crosses prompted us to investigate the interference of YB-1 with topoisomerase II. To that end, we used kinetoplast (kDNA) which is a network of many circular DNAs. Topoisomerase II, but not topoisomerase I, has the ability to release circular DNAs from this network in a process called decatenation. We thus analyzed the conversion rate of kDNA into decatenated DNA circles mediated by topoisomerase II (Figure 7A). The results indicate that YB-1 significantly decreases the topoisomerase II activity from nuclear NRK cell extracts. In contrast, G3BP, used as control, does not prevent the topoisomerase II activity (Figure 7B). To further probe the relevance of the inhibition of the topoisomerase activity by YB-1, we used PARP-1, a DNA-binding protein which also displays a higher affinity for DNA crosses (57). We found that, in contrast with YB-1, PARP-1 is not an obstacle for the decatenation of kinetoplast DNA. As previously reported, PARP-1 may inhibit the topoisomerase activity but only when activated in the presence of NAD^+^ to produce Poly (ADP-ribose) (58). In the case of YB-1, its multimerization at DNA crosses could be an efficient mechanism to prevent the binding of topoisomerase II. In agreement with this, YB-1 does form multimers at crosses between catenated DNA circles, as revealed by AFM (Figure 7C). This result indicates that YB-1 and topoisomerase II may compete for the binding to DNA crosses. In addition, YB-1 does not progressively reduce topoisomerase activity but does it rather abruptly above a critical concentration of YB-1, which may reflect the multimerization of YB-1 above a threshold concentration.

## DISCUSSION

### Protein multimerization as a novel mechanism for selective translational regulation

**Figure 8.**
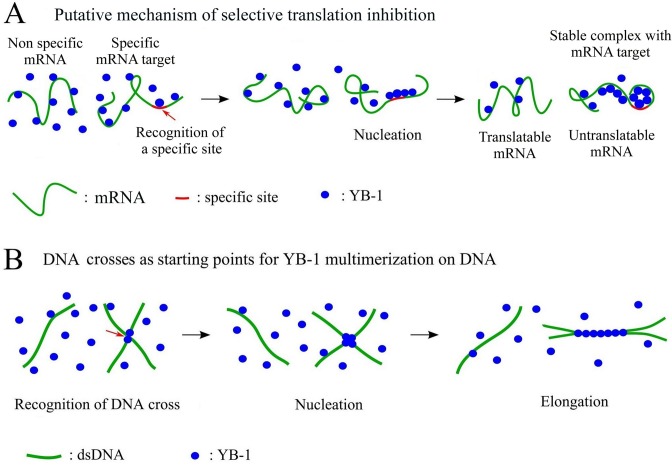
Schematic view of the selective binding of YB-1 to specific mRNA and DNA crosses. (**A**) Due to the cooperative binding of YB-1 to mRNA, a preferential multimerization on specific mRNAs could take place due to the presence of specific sites. As the formation of YB-1 multimers on specific mRNA decreases the pool of free YB-1, the chances of YB-1 multimerization on non-specific mRNAs are reduced. At the end of the process, YB-1 only inhibits the translation of specific mRNAs. (**B**) When interacting with rigid dsDNA polymer, YB-1 proteins are well separated from each other, which precludes YB-1 multimerization. The interface between two DNA helices serves as nucleation site for protein multimerization. YB-1 multimerization along two DNA helices forces them to adopt a parallel trajectory.

Many mRNA-binding proteins display a weak specificity toward short and redundant RNA sequences/structures, *in vitro*, while having for many of them a significant selectivity toward specific mRNA in the cellular context (59). RNA selection by dedicated proteins is therefore the focus of ongoing investigations and, except for some RNA-binding proteins displaying a strong affinity for specific sequences (60), the molecular mechanisms at the heart of such selective binding remain elusive (22,61,62). In addition, as recently shown with the RNA-binding protein C5 (61), the selectivity is not necessarily driven by a strong affinity and case-by-case studies may be the key to unravel the probable various mechanisms adopted by RNA-binding proteins for an efficient selectivity. In light of the present results, we advance a novel mechanism leading to selectivity among transcripts using YB-1 as a model: a preferential protein multimerization based on a cooperative binding to mRNA (Figure 8A). YB-1 is a possible protein candidate to test this model because it possesses some critical characteristics for an efficient multimerization on mRNA. The cold-shock domain of YB-1 has a low affinity for RNA and can form multimers in solution (Figure 2 and S4). In the absence of RNA, the highly positively charged C-terminus of YB-1 is an obstacle to YB-1 multimerization via its cold-shock domain (Figure S4). However, when bound to negatively-charged mRNA, the electrostatic repulsion due the C-terminus of YB-1 is no longer effective, which enables YB-1 multimerization on mRNA (Figure S2B). In agreement with this, the experimental results indicate that the cold-shock domain plays a critical role for the cooperative binding of YB-1 to mRNA and the ensuing YB-1 multimerization (Figures 2, 3 and S2B).

If we assume that the residence time of YB-1 on specific RNA sites is longer than average, the chance to attract other YB-1 molecules via YB-1 self-attraction increases accordingly. Once YB-1 multimerization starts, the energy benefit in attracting others YB-1 molecules significantly increases because there are many YB-1 molecules on mRNA to interact with them. Owing to the energy benefit of YB-1 multimerization, a permanent regime may be reached where some mRNAs, which have preferential nucleation sites, form saturated YB-1:mRNA complexes while YB-1 is apparently kept at bay from non specific mRNAs. In a sense, the specificity of the initial nucleation is detected, amplified and energy-secure via a cooperative binding. A related mechanism has also been proposed to explain the specific packaging of viral RNA by proteins via a cooperative binding (63).

As a proof of concept of this model, we show that the cooperative binding of YB-1 to mRNA stabilizes saturated YB-1:mRNA complexes (Figure 4C). In addition, after mixing saturated complexes with other mRNAs, we obtained a preferential translation repression of the pre-incubated mRNAs compared to the other coexisting mRNAs in rabbit reticulocyte system. We therefore succeeded in manipulating translation selectively (Figure 4D).

At first sight, such model of protein multimerization on specific mRNAs could be challenged by other competing mRNA-binding proteins *in vivo*. To address this issue, we show that the presence of G3BP, a non-cooperative mRNA-binding protein, was not an obstacle for the cooperative binding of YB-1 to mRNA (Figure 4A and B). The point is that YB-1 multimerization most probably provides a huge energy benefit which enables to displace isolated RNA-binding proteins like G3BP.

Other RNA binding-proteins than YB-1 could orchestrate translation repression via a similar mechanism as protein self-attraction is an over-represented property among RNA-binding proteins, mostly due the high occurrence of low complexity sequences or prion-like domains (64–66). In line with this, multimerization on RNA-protein target has already been described for other RNA-binding proteins (65–70) like HuR and FUS. It is thus of interest to explore whether a similar mechanism of translation repression can take place for these proteins.

Finally, an unanswered issue, which applies to other selective translation mechanisms orchestrated by RNA-binding proteins, is whether the affinity of RNA-binding protein for short mRNA sequences results in a selective binding to mRNA. Indeed, while such preferential binding is well documented for short hexa/hepta nucleotides *in vitro* (22), it remains elusive for long mRNA molecules. The presence of many non-specific RNA sites in long mRNA probably hinders the preferential binding to specific sequences. *In vitro* approaches may require additional protein factors that may be lacking so far. For example, the initial recruitment of proteins like YB-1 may rely on interactions with other mRNA-binding proteins having a high affinity for specific mRNA sequences/structures, as proposed for DDX6 (71). These issues deserve to be considered in the future to further document the mechanisms of selective translation.

### YB-1 binding to DNA and putative functions in DNA-related processes

So far, the proposed nuclear functions of YB-1 related to its interactions with DNA were based on its reported preferential binding to damaged DNA (36) and, possibly, to the promoter regions of many genes implicated in cell proliferation, multi-drug resistance or DNA repair (31). Albeit interesting, major concerns have been raised regarding the putative function of YB-1 as a transcription factor (32) based on its weak affinity to specific DNA sequences. Similarly, the molecular mechanism by which YB-1 participates to DNA repair despite the presence of many other DNA-binding proteins of higher affinity toward DNA than YB-1 remains to be clarified.

Here we advanced an alternative view which allows YB-1 to become a relevant competitor for the binding to DNA. YB-1 should not be considered as an isolated protein with a strong affinity to specific DNA structures but rather as a protein able to multimerize at specific DNA sites. The energy benefit in forming YB-1 multimers on DNA results from the sum of the YB-1:DNA and YB-1:YB-1 interactions, which should clearly reduce the chance of being displaced from DNA by other proteins. In line with this, we found a striking preferential multimerization of YB-1 at the interface between two parallel DNA helices starting from DNA crosses (Figure 5A and B). The difference in the persistence length between mRNA and DNA may provide a rational explanation for the preference of YB-1 for mRNA and DNA crosses compared to relaxed DNA (Figure 8B). The number of nucleotides separating two consecutive YB-1 molecules on mRNA is large (about 30–40 nucleotides), which roughly corresponds to the number of phosphate charges sufficient to neutralize the clusters of positive residues located in the YB-1 C-terminus (net charge: +41 e^−^). Despite this limitation, mRNA flexibility can allow many YB-1 molecules to interact physically into globules (Figure 8A). DNA rigidity prevents such short-ranged bending. As a result, on isolated DNA, YB-1 cannot form multimer which would require an impossible DNA bending. On the other hand, at DNA crosses, YB-1 molecules can interact with each other at the interface between two DNA helices without mechanical constraint (Figure 8B). In agreement with this view, we show that YB-1 prefers to multimerize along supercoiled plasmids than on relaxed plasmids or linear DNA because the occurrence of crosses is significantly higher in supercoiled DNA (Figure 5B and S7).

If we now consider what could be the possible consequences of the YB-1 multimerization at DNA crosses and the ensuing alignment of DNA helices (Figure 8B), our attention then naturally turns to topoisomerase II which binds to DNA at crosses to modify supercoiled DNA topology. As observed from NRK cell extracts (Figure 7), YB-1 interferes negatively with the topoisomerase II activity, most probably by masking DNA crosses. The expression of topoisomerase II correlates with the presence of YB-1 in the nucleus which has been attributed to a putative targeting of an inverted CCAAT box in the promoter region of topoisomerase II (30). However, the role of YB-1 in transcription is challenged by recent reports (40,72), YB-1 may rather indirectly increase toposiomerase II expression level to compensate the YB-1-mediated loss of topoisomerase II activity. In addition to this, YB-1 may also participate/interfere with some DNA repair mechanisms like homologous recombination (73) through the YB-1-mediated alignment of two DNA helices (Figure 6D). Altogether, our mechanistic view of the binding of YB-1 to DNA may provide a novel view on its role in DNA repair and drug resistance of cancer cells.

## Supplementary Material

SUPPLEMENTARY DATA
