# Supplementary material for: mRNA and DNA selection via protein multimerization: YB-1 as a case study
Source: Nucleic Acids Res. 2015 Aug 13;43(19):9457–73. doi: 10.1093/nar/gkv822 (PMC4627072; doi:10.1093/nar/gkv822)
Supplement: SUPPLEMENTARY DATA [file supp_43_19_9457__index.html]

mRNA and DNA selection via protein multimerization: YB-1 as a case study — mRNA and DNA selection via protein multimerization: YB-1 as a case study — mRNA and DNA selection via protein multimerization: YB-1 as a case study — SUPPLEMENTARY DATA 

# mRNA and DNA selection via protein multimerization: YB-1 as a case study

## SUPPLEMENTARY DATA

- SUPPLEMENTARY DATA
