# Supplementary material for: mRNA and DNA selection via protein multimerization: YB-1 as a case study
Source: Nucleic Acids Res. 2015 Aug 13;43(19):9457–73. doi: 10.1093/nar/gkv822 (PMC4627072; doi:10.1093/nar/gkv822)
Supplement: SUPPLEMENTARY DATA [file supp_gkv822_nar-01840-z-2015-File002.docx]

**
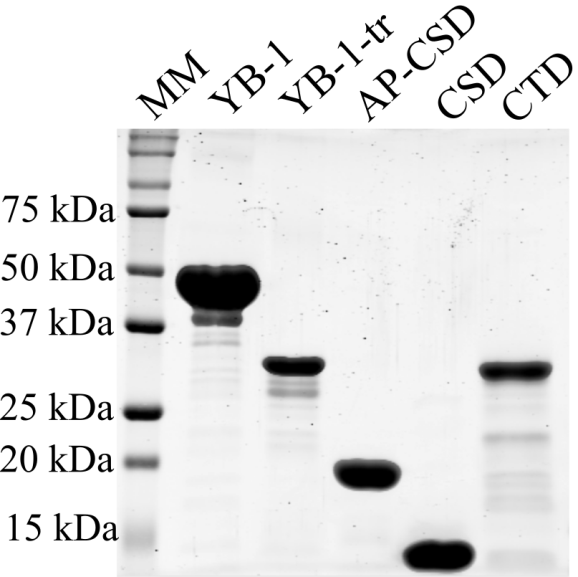

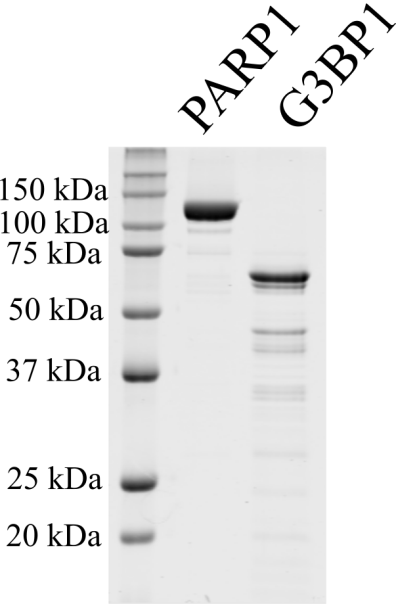
**

Figure S1: Purified recombinant proteins used in this study, as observed after their migration in a denaturating gel.

****A)

2 Luc mRNA

B)

Coomassie blue

(Protein)

Ethidium bromide

(RNA or DNA)

Figure S2: The cooperative binding of YB-1 to mRNA depends on ionic strength. Cross-linking indicates that YB-1 multimerizes upon binding to RNA (2Luc mRNA) and DNA (pBR322).

1. Gel mobility shift assays reflecting the influence of the ionic strength on the cooperative binding of YB-1 to 2Luc mRNA were performed under indicated concentrations of KCl. The appearance of two distinct migration bands is clearly detected at moderate ionic strength (50 mM KCl). At 250 mM KCl, YB-1 cooperativity is no longer detected as only a single migration band is observed. mRNA: 0.25 pmoles per well.
2. In the absence of RNA or DNA, YB-1 is mostly in its monomeric state after its crosslinking by glutaraldehyde. On the other hand, in the presence of both DNA and RNA, we noticed the appearance of large YB-1-rich and DNA or RNA-rich bodies which cannot enter into the gel being observed at the bottom of the well. The addition of RNAse or DNAse after crosslinking fails to dissociate the YB-1 multimers. These results indicate that direct YB-1:YB-1 interactions take place on both DNA and RNA which serve as scaffolds for the multimerization process. YB1-1:5 µg per well. DNA: 1 µg per well. RNA: 1 µg per well.


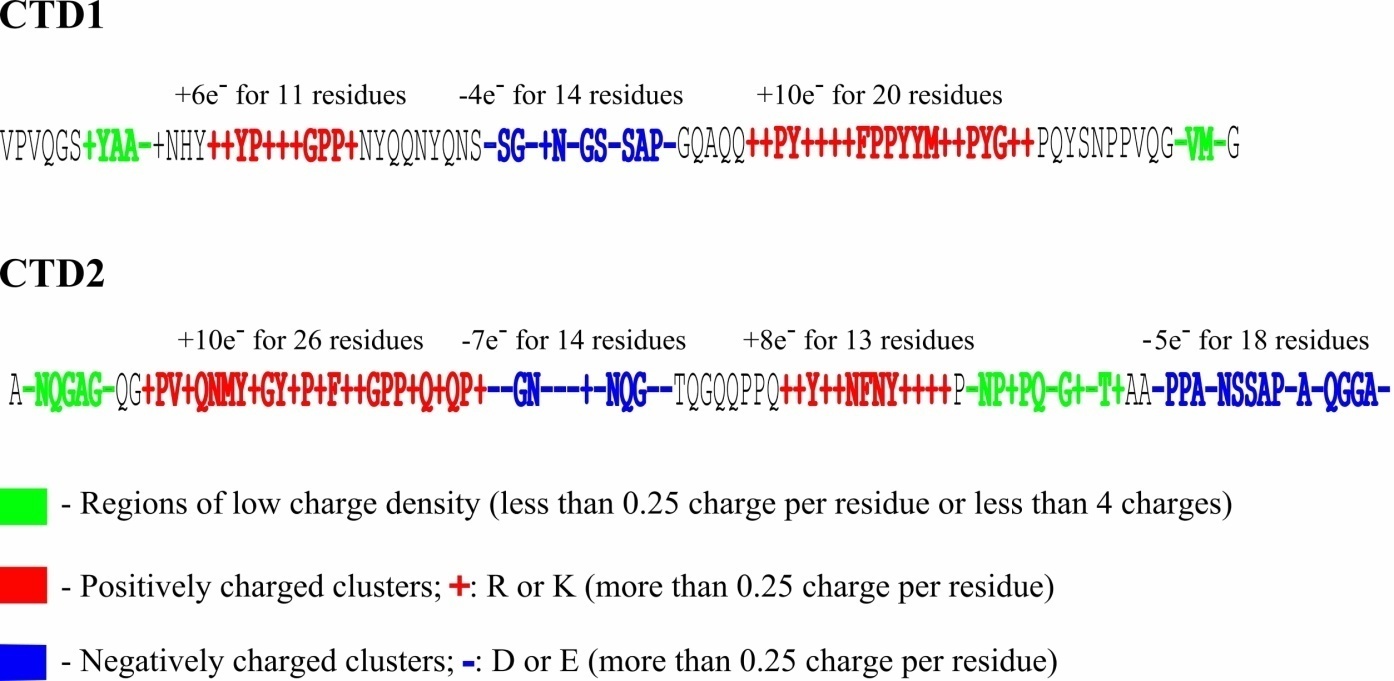


Figure S3: Analysis of the charge distribution of CTD1 and CTD2.

1. CTD1 is mostly positively charged (net charge: +12 e^-^) and displays two positively charged clusters containing 6 and 10 positive charges. Their average charge density is +0.54 and +0.5e^-^ per residues is also significant. In contrast, the only putative cluster of negative charge of CTD1 contains only 4 net negative charges and a low charge density (- 0.29 e^-^ per residues). In the presence of highly negatively charged RNA, we can reasonably assume that the weakly negatively charged cluster cannot compete with RNA for the binding to the two positive clusters of CTD1.
2. CTD2 possesses two positively charged clusters (+10 and +8 e^-^) and two negative clusters (-7 and -5 e^-^) which may lead to an electrostatic attraction to the positively charged clusters (electrostatic zipper).

**** A)


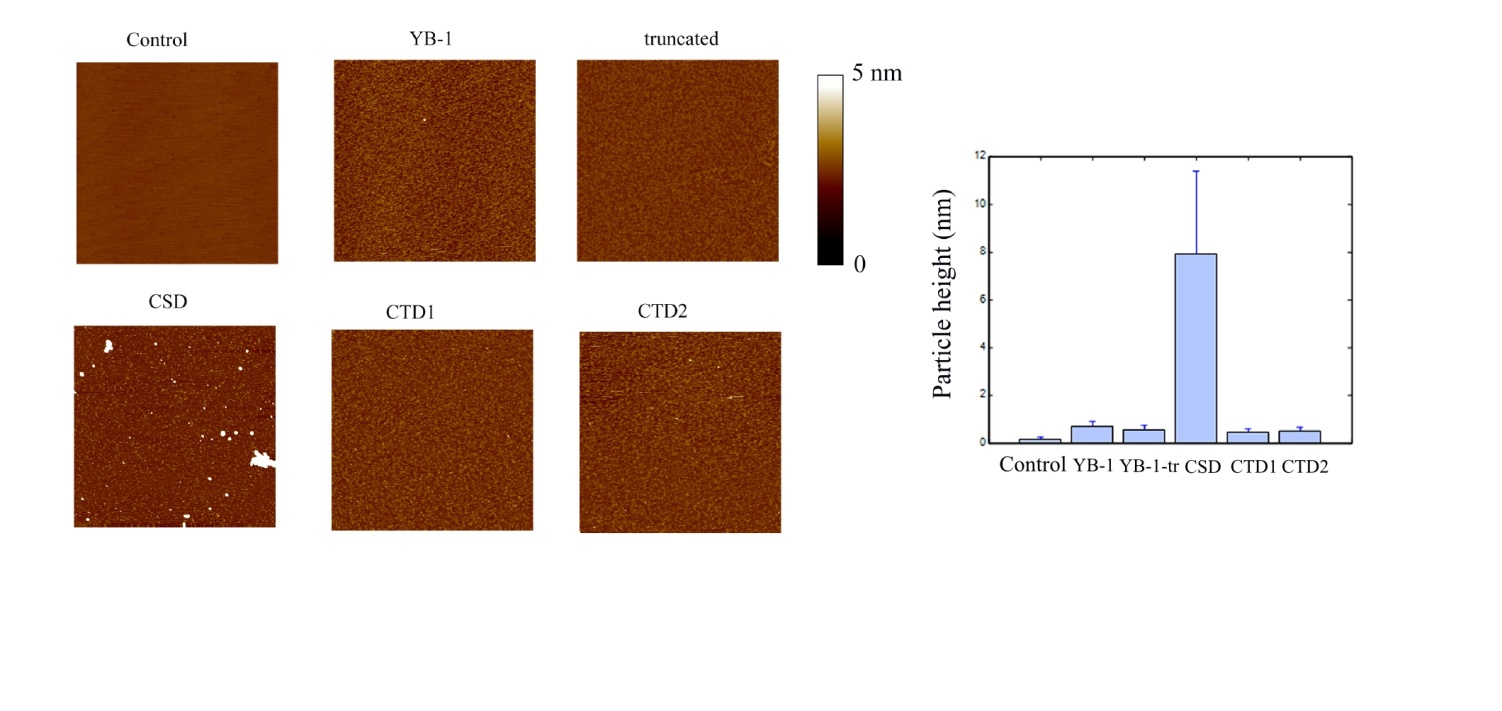
B)

Figure S4: CSD but not CTD form multimers *in vitro*.

1. Protein-protein interactions were detected for the indicated YB-1 constructs (0.5 mg/ml) using 0.1 % Glutaraldehyde for 10 min at room temperature. APCSD is a construct with A/P and CSD domains (N-terminal terminus of YB-1). A significant multimerization was only detected in the presence of CSD or APCSD while YB-1, YB-1-tr and CTD mostly remain in their monomeric state when free in solution. The presence of the CTD in latter constructs may be responsive for a self-repulsion of electrostatic origin and thus prevents their self-attraction.
2. AFM images of YB-1 constructs by atomic force microscopy reveal the formation of multimolecular complex in the presence of CSD but not for CTD. Same conditions as A) without glutaraldehyde: Incubation time: 30 min.

A)

Significant

B)

Not significant

Figure S5: Analysis of the height distributions and determination of two statistically different populations. The height measurements obtained by AFM in a given condition were arbitrary separated in two populations using a varying critical height. The two populations correspond to height data points having a lower or larger height than the critical height. The plot represents the mean heights and the standard deviations of the two populations versus the critical height. We then determined for which critical height the two populations were significantly different, if any.

A) The statistical analyze indicate that the two populations were statistically different with the highest significance for a critical height of about 3.3 nm (dashed ellipse), which give two populations: 3.98 ± 0.46 nm and 2.3 ± 0.4 nm (figure 4B).

B) Whatever the critical height, the two populations were not statistically different. We then considered that only one population exists (1.8 ± 0.33 nm, figure 3B with CTD).

**A**

0 20 40 80

**B**

Figure S6: YB-1 does not selectively inhibit translation without the pre-incubation step.

1. In vitro translation assays in rabbit reticulocyte system of a mixture of Luc mRNA (~1500 nt, 0.25 pmoles) and HA-YB-1 mRNA (~1500 nt, 0.25 pmoles) at varying concentration of YB-1. YB-1 was directly added to the equimolar mixture of Luc and YB-1 mRNA. We observed that the translation repression exerted by YB-1 is similar for both mRNAs.
2. Anti-YB-1 was used to control the concentration of YB-1 under the conditions tested in figure 4B.

We noticed the presence of both endogenous YB-1 from the rabbit reticulocyte extracts and HA-YB-1 (see arrow). We also noticed the gradual increase of the recombinant YB-1 concentration which exceeds that of endogenous YB-1 and HA-YB-1, which was a prerequisite to analyze translation inhibition mediated by YB-1.

Figure S7: YB-1 and YB-1-tr multimerization at DNA crosses leads to its selective binding to supercoiled DNA.

### The interactions of YB-1, YB-1-tr and G3BP (300 nM) with an equimolar mixture containing linear 1220 bp and supercoiled pBR322 DNA (2 nM each) were observed on mica by AFM. In the presence of YB-1 and YB-1-tr, YB-1 multimerization is detected exclusively on supercoiled DNA. Such pattern is not observed for G3BP used as control. The higher magnification image clearly highlights the highly selective binding of YB-1 to supercoiled DNA at DNA crossovers. Scale bar: 100 nm.

1. B)


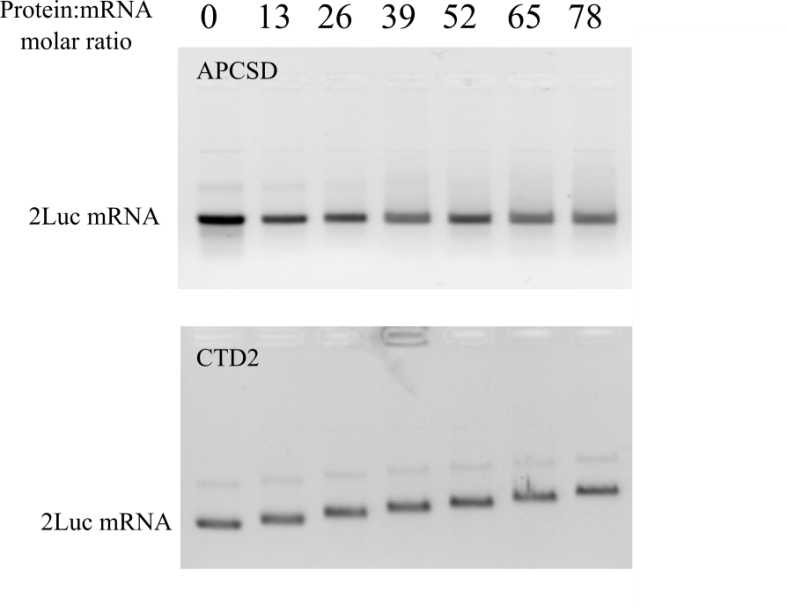


Figure S8: CTD2 preferentially binds to supercoiled DNA while the binding of AP-CSD to mRNA or supercoiled and relaxed DNA is not detected.

1. Gel mobility shift assay probing the binding of YB-1 of an equimolar mixture of supercoiled and nicked pBR322 DNA (70 fmoles). The quantity of protein ranged from 3 to 21 pmoles for both constructs. As CTD, AP-CSD (A/P +CSD domains) is not binding DNA significantly *in vitro,* which indicates that the A/P domain does not reinforce the binding of CSD to DNA. CTD2 shows a preferential binding to supercoiled DNA, most probably by forming a salt-bridge at the interface between two DNA helices.
2. Gel mobility shift assay reflecting the binding of AP-CSD and CTD2. AP-CSD does not significantly reduce the mobility of mRNA. Similar results were obtained with the CSD alone. Increasing CTD2 leads to a gradual decrease of the nucleoprotein complex mobility.
